# [^18^F]fluciclovine vs. [^18^F]fluorocholine Positron Emission Tomography/Computed Tomography: A Head-to-Head Comparison for Early Detection of Biochemical Recurrence in Prostate Cancer Patients

**DOI:** 10.3390/tomography8060226

**Published:** 2022-11-05

**Authors:** Cristina Ferrari, Paolo Mammucci, Valentina Lavelli, Antonio Rosario Pisani, Anna Giulia Nappi, Dino Rubini, Angela Sardaro, Giuseppe Rubini

**Affiliations:** 1 Interdisciplinary Department of Medicine, University of Bari Aldo Moro, Piazza Giulio Cesare 11, 70124 Bari, Italy; 2 Section of Radiology and Radiation Oncology, Interdisciplinary Department of Medicine, University of Bari Aldo Moro, Piazza Giulio Cesare 11, 70124 Bari, Italy

**Keywords:** prostate cancer, PET/CT, [^18^F]fluciclovine, [^18^F]fluorocholine, biochemical recurrence, PSA

## Abstract

Nowadays, there is still no consensus on the most accurate PET radiopharmaceutical to early detect prostate cancer (PCa) relapse. A tailored radiotracer choice based on a specific patient’s profile could ensure prompt disease detection and an improvement in patients management. We aimed to compare the [^18^F]fluciclovine and [^18^F]fluorocholine PET/CT detection rate (DR) in PCa patients restaged for early biochemical recurrence (BCR), according to clinical and biochemical features. A cohort of 138 PCa patients with early BCR (mean age: 71 y, range: 50–87 y) were homogeneously randomized 1:1 to a [^18^F]fluciclovine or a [^18^F]fluorocholine PET/CT group. The respective PET/CT DR, according to per-patient and per-region analysis, and the impact of the biochemical, clinical, and histological parameters, were compared. The PSA cut-off values predictive of a positive scan were also calculated. Overall, the [^18^F]fluciclovine PET/CT DR was 64%, significantly higher than the [^18^F]fluorocholine PET/CT DR of 35% (*p* = 0.001). Similarly, in the per-region analysis, the [^18^F]fluciclovine PET/CT DR was 51% in the prostate region, significantly higher compared to 15% of [^18^F]fluorocholine (*p* < 0.0001). Furthermore, a statistically significant higher DR in per-patient and per-region (prostate/prostate bed) analysis was observed in the [^18^F]fluciclovine group for 0.5–1 ng/mL (*p* = 0.018, *p* = 0.049) and >1 ng/mL (*p* = 0.040, *p* < 0.0001) PSA values. A PSA of 0.45 ng/mL for [^18^F]fluciclovine and of 0.94 ng/mL for [^18^F]fluorocholine was identified as the optimal cut-off value in predicting a positive PET/CT scan. Our results demonstrated a better [^18^F]fluciclovine PET/CT DR compared to [^18^F]fluorocholine for restaging PCa patients in early BCR, particularly in the detection of locoregional recurrence. The significantly higher [^18^F]fluciclovine DR for low PSA values (PSA < 1 ng/mL) supports its use in this setting of patients.

## 1. Introduction

According to the most recent GLOBOCAN 2020 database, prostate cancer (PCa) remains one of the most common tumors and the fifth leading cause of cancer-related death among men, with a worldwide estimation of 1.4 million diagnoses in 2020 and a prevalence of 59% (48–71%) [1].

Despite improvements in radical treatment with curative intent in clinically localized PCa, the risk of recurrence still occurs in 20–50% after radical prostatectomy (RP) and in 30–40% after radiotherapy (RT) [2,3].

After primary treatment, biochemical parameter testing remains a sensitive method to monitor PCa disease and identify biochemical recurrence (BCR). A disease relapse could be revealed by prostate-specific antigen (PSA) level increase, and characterized by PSA kinetics, particularly PSA doubling time (PSAdt) [4].

Once BCR is diagnosed, a non-invasive imaging technique is crucial to early detect local and/or systemic tumor recurrence, guiding the most appropriate therapeutic strategy.

Common imaging methods for investigating disease recurrence suspicion include bone scintigraphy using hydroxymethylene diphosphonate labeled with ^99m^Tc, abdominopelvic computed tomography (CT), and magnetic resonance imaging (MRI). However, all these techniques often fail to early localize tumor relapse, particularly in asymptomatic patients with low PSA values [5].

In this scenario, Positron Emission Tomography/Computed Tomography (PET/CT) has proved to be an extremely reliable technique for evaluating PCa patients with BCR.

While [^18^F]fluorodeoxyglucose (FDG) PET/CT has a well-established role in diagnosing, staging, restaging, and treatment response assessment of different oncological fields, consolidated evidence suggests its limited usefulness in PCa patients [6,7].

After Food and Drug Administration (FDA) approval in 2012, choline labeled with Fluorine-18 or Carbon-11 became the most widely used PET radiopharmaceutical for PCa patients. In BCR restaging, it demonstrated a sensitivity and specificity ranging from 86 to 89% and from 89 to 93%, respectively, higher than stand-alone morphologic imaging (MRI or CT) [8]. However, the clinical utility of choline-labeled PET/CT remains controversial in patients with early BCR, because its detection rate (DR) and sensitivity are strongly dependent on PSA level and kinetics [9].

The need for a more accurate PET radiopharmaceutical in BCR patients with low PSA value led to a growing interest in researching new and performing tracers for this particular setting of patients, including [^18^F]fluciclovine and radiolabeled prostate-specific membrane antigen (PSMA).

Differently from PSMA, which has a receptor mechanism, both fluciclovine and choline present an intracellular uptake [10].

[^18^F]fluciclovine is a synthetic amino acid absorbed via the L-type amino acid transporter (LAT1) and the sodium-dependent neutral amino acid transporter (ASCT2), up-regulated in many human cancers, including PCa [11].

It is recommended for restaging PCa patients in BCR following primary radical treatment and its performance showed promising results also in the case of low PSA levels [9].

Nowadays, there is still no consensus on the most accurate PET radiopharmaceutical to early detect PCa recurrent lesions. Even if [^18^F]fluorocholine PET/CT has a well-established role in PCa patients, only a few studies focus on a head-to-head comparison between [^18^F]fluciclovine and [^18^F]fluorocholine PET/CT diagnostic performance.

Our study aims to compare the [^18^F]fluciclovine and [^18^F]fluorocholine PET/CT DR in PCa patients with early BCR, according to biochemical, clinical, and histological features.

## 2. Materials and Methods

### 2.1. Study Design

Between September 2019 and February 2022, a PCa patients’ cohort was restaged for BCR with a prostate-dedicated radiopharmaceutical PET/CT.

The inclusion criteria were: (1) adult male above 18 years; (2) proven PCa treated with RP, with or without adjuvant RT, or with primary RT; (3) proven BCR with rising PSA levels (PSA ≥ 0.2 ng/mL after RP, PSA ≥ 2 ng/mL above the nadir after primary RT); and/or (4) clinical suspicious of disease recurrence (symptomatic patients).

Patients enrolled in this single-center study were randomized 1:1 to a [^18^F]fluciclovine or a [^18^F]fluorocholine group, using a computer-generated randomization schedule with an algorithm that ensures a homogeneous PSA distribution.

During anamnesis, the physician collected clinical information, including primary treatment (RP, RT), histological features (stage and initial Gleason Score, GS), previous imaging investigations, previous and ongoing therapies (hormonal therapy, HT), as well as previous and current PSA values at the time of the scan. The collected data were used to calculate PSAdt (https://www.mskcc.org/nomograms/prostate/psa_doubling_time (accessed on 1 October 2022)) and to classify patients according to the new European Association of Urology (EAU) BCR risk group [12,13]. Based on literature data and international guidelines, PCa patients were stratified according to GS (<8 or ≥8), EAU BCR risk group (low or high), PSA value (<0.5, 0.5–1 or >1 ng/mL), PSAdt (≤12 months or >12 months), and ongoing HT (yes vs. no) [5].

Written informed consent was obtained for all the enrolled patients. The present study was approved by the local Ethics Committee (Prot. n. 0012052|08/02/2022|AOUCPG23|COMET|P) and performed in accordance with the principles of the Declaration of Helsinki and with national regulations.

### 2.2. Imaging Protocol and Analysis

All PET/CT examinations were performed in accordance with the European Association of Nuclear Medicine (EANM) guidelines [14]. No physical exercise the day before the exam and fasting for at least 4 h before injection of [^18^F]fluciclovine were required; additionally, patients were asked not to void 30–60 min before imaging. A dose of approximately 370 MBq of [^18^F]fluciclovine and 3–4 MBq/kg body weight (range 240–390 MBq/kg) of [^18^F]fluorocholine was intravenously administered. All scans were obtained using a hybrid PET/CT scanner (Discovery 710, GE, General Electrics, Milwaukee, WI, USA). After 5 min post-injection, early [^18^F]fluorocholine PET/CT images of the pelvis were acquired targeting the prostate area, followed by a whole-body image acquisition after 60 min, from the skull base to the proximal third of the femurs. To perform the [^18^F]fluciclovine PET/CT exam, immediately after the intravenous radiopharmaceutical administration, a low dose CT scan without contrast agents started, followed by a PET acquisition at 3–5 min post-injection, from the mid-thigh to the base of the skull (5–6 bed positions). A 3D acquisition mode PET scan was performed for the same longitudinal coverage (2.5 min per bed position). Co-registered CT parameters were as follows: pitch 0.98, gantry rotation speed of 0.5 s/rot, 120 kV, and modulated tube current of 140 mA. CT images were used both for image fusion and anatomical localization and for attenuation correction of emission data.

### 2.3. Image Analysis

Image analysis was carried out using a dedicated console (AW Server 4.7, General Electrics, Milwaukee, WI, USA). PET/CT scans were independently evaluated by two nuclear medicine physicians with at least 3 years of experience in [^18^F]fluciclovine and [^18^F]fluorocholine PET/CT reading and aware of clinical data. In the event of disagreement, a final consensus was reached.

Maximum intensity projection (MIP), PET, CT, and PET/CT fused images in different planes (axial, sagittal, and coronal) were visualized simultaneously to correctly interpret scans. Examinations were considered positive in the presence of focal areas of detectable increased tracer uptake, visually more intense than the background, not correlating with physiological tracer uptake and inflammatory articular processes, with or without any underlying lesion identified on the co-registered CT [15,16]. The semiquantitative evaluation by the maximum standardized uptake value (SUV) and, for [^18^F]fluciclovine PET/CT image, also by the SUV ratio (SUVmax in the lesion/SUVmean in the surrounding background) was used to aid visual analysis. In particular, bone marrow uptake of vertebra L3 was used as a reference for lesions larger than a 1 cm longest dimension, and abdominal aortic blood pool for lesions smaller than a 1 cm longest dimension [14].

### 2.4. Data Analysis

[^18^F]fluciclovine and [^18^F]fluorocholine PET/CT-positive findings were reported as “detection rate”, defined as the proportion of scans containing one or more areas considered positive for cancer since histological confirmation was not available or feasible. The respective PET/CT DR were compared, according to per-patient and per-region analysis (prostate/prostatectomy bed, lymph node, bone).

In addition, the impact of biochemical parameters (PSA value, PSAdt), and clinical-histological variables (GS, EAU BCR risk group, ongoing HT) on the [^18^F]fluciclovine and [^18^F]fluorocholine PET/CT DR was assessed, and then compared.

The PSA cut-off values predictive of a positive [^18^F]fluciclovine and [^18^F]fluorocholine scan were calculated.

Finally, focusing on positive PET scans, the number of lesions in per-patient and per-region analyses and the impact of PSA level and PSAdt were compared between the two radiopharmaceuticals groups.

### 2.5. Statistical Analysis

Quantitative variables were expressed as mean with standard deviation. Student t-tests provided an estimate of the difference between [^18^F]fluciclovine and [^18^F]fluorocholine PET/CT arms.

Categorical variables were presented with absolute and relative frequencies. [^18^F]fluciclovine and [^18^F]fluorocholine PET/CT per-patient and per-region DR were compared by using the Chi-square and Fisher exact test. The same tests were employed to analyze the DR difference between the two groups in relation to the following categorical variables: PSA value (<0.5, 0.5–1, >1), PSAdt (≤12 months, >12 months), GS (<8, ≥8), EAU BCR risk group (low-, high-risk), ongoing HT (yes vs. no). The Mann–Whitney U test was used to compare differences between continuous non-normally distributed variables (PSA values; the number of recurrence lesions).

The performance of [^18^F]fluciclovine and [^18^F]fluorocholine PET/CT in relation to the PSA value was assessed by the receiving-operating-characteristic (ROC) curves generated by plotting sensitivity versus 1—specificity. The best PSA cut-off value for predicting a positive [^18^F]fluciclovine and [^18^F]fluorocholine PET/CT scan was determined by Youden’s index. Statistical significance was assumed for *p*-values less than 0.05.

All statistical analyses were performed using SPSS statistical software, version 25 (IBM Corporation, Armonk, NY, USA).

## 3. Results

According to the inclusion criteria, 138 PCa patients (mean age: 71 y, range: 50–87 y) were enrolled in our study and randomized 1:1 to the [^18^F]fluciclovine (*n* = 69) or the [^18^F]fluorocholine (*n* = 69) group, resulting homogeneously for PSA value, PSA kinetics and clinical-histological features.

Patients’ characteristics are described in detail in Table 1.

### 3.1. Per-Patient and Per-Region Detection Rate

According to the per-patient analysis, the [^18^F]fluciclovine PET/CT DR was 64% (44/69), significantly higher compared to the [^18^F]fluorocholine PET/CT DR of 35% (24/69) (*p* = 0.001).

**Figure 1:**
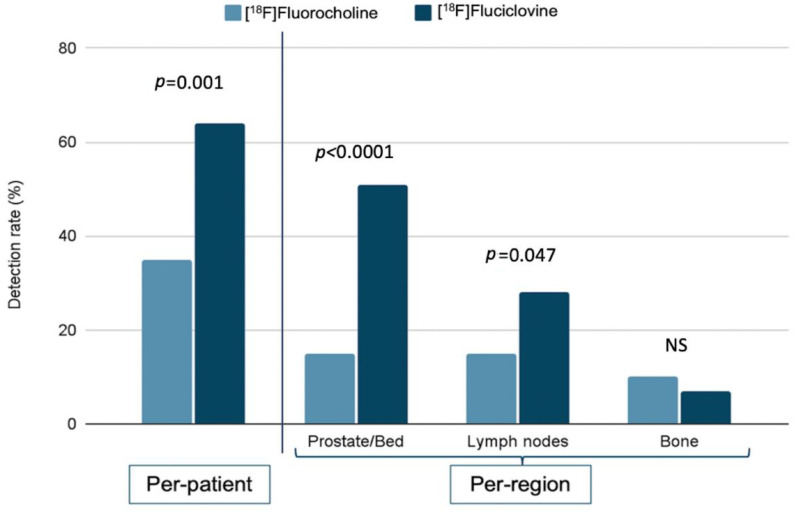
[^18^F]fluorocholine and [^18^F]fluciclovine PET/CT detection rate according to per-patient and per-region analysis. NS: non-significant *p*-value.

Analogously, in the per-region analysis, the [^18^F]fluciclovine PET/CT DR in prostate/prostatectomy bed region was 51% (35/69), resulting significantly higher than the [^18^F]fluorocholine group (15%, 10/69) (*p* < 0.0001). The two radiotracers showed a weakly significant difference in the lymph node DR, namely the lymph node region resulted positive in 28% (19/69) of [^18^F]fluciclovine patients (*p* = 0.047) and in 15% (10/69) of [^18^F]fluorocholine patients. No statistically significant difference was found in the bone lesions DR, corresponding to 7% (5/69) and 10% (7/69) for the [^18^F]fluciclovine and [^18^F]fluorocholine group, respectively (*p* = 0.382) (Figure 1).

### 3.2. Biochemical Parameters

In both the [^18^F]fluorocholine and [^18^F]fluciclovine groups, the PSA value impacted the PET/CT DR. For the growing PSA values (<0.5, 0.5–1, and >1), increasingly higher percentages of positive [^18^F]fluorocholine PET/CT scan were observed with a DR of 16% (4/25), 31% (5/16) and 54% (15/28), respectively (*p* = 0.016). A growing DR was also observed in the [^18^F]fluciclovine PET/CT group, as 43% (10/23), 61% (14/23), and 87% (20/23), respectively (*p* = 0.009).

Comparing the two radiopharmaceuticals groups, a statistically significant higher per-patient DR was observed in the [^18^F]fluciclovine group for 0.5–1 ng/mL (*p* = 0.018) and >1 ng/mL (*p* = 0.040) PSA levels, as reported in Table 2. This result was confirmed in the per-region analysis, especially in the prostate/prostatectomy bed (PSA 0.5–1 ng/mL: +32%, *p* = 0.049; PSA >1 ng/mL: +57%, *p* < 0.0001).

Bone was the only region with a higher DR in the [^18^F]fluorocholine group for PSA > 1 ng/mL level (*p* > 0.05).

**Figure 2:**
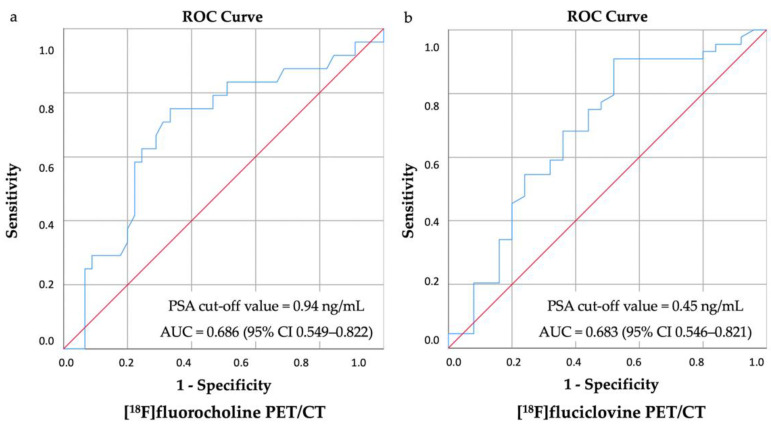
ROC analysis identified the optimal PSA cut-off value of (**a**) 0.94 ng/mL for the [^18^F]fluorocholine PET/CT and of (**b**) 0.45 ng/mL for the [^18^F]fluciclovine PET/CT.

ROC analysis showed a PSA of 0.45 ng/mL (sensitivity 87%, specificity 50%) for the [^18^F]fluciclovine group (AUC = 0.693, 95% CI 0.554–0.834) and of 0.94 ng/mL (sensitivity 75%, specificity 67%) for the [^18^F]fluorocholine group (AUC = 0.686, 95% CI 0.548–0.824) as the optimal cut-off values in predicting a positive PET/CT scan (Figure 2).

PSAdt did not show a statistically significant impact on the per-patient [^18^F]fluciclovine PET/CT DR. Conversely, PSAdt impacted the [^18^F]fluorocholine PET/CT DR, namely a significantly higher number of positive scans was observed in patients with a faster PSAdt (PSAdt ≤ 12 months: 55%, 12/22; PSAdt > 12 months: 21%; *p* = 0.018).

For PSAdt, the comparison between the two radiopharmaceuticals groups showed a statistically significant higher overall DR in the [^18^F]fluciclovine group for a slower PSAdt value (*p* = 0.001). All per-patient DR results are reported in Table 3.

### 3.3. Clinical and Histological Parameters

Comparing the two radiopharmaceuticals groups, a statistically significant higher DR was identified in the [^18^F]fluciclovine one for GS <8, low EAU BCR Risk Group, and in the absence of an ongoing HT. Table 4 reported all clinical-histological analysis results.

### 3.4. Recurrent Lesions’ Number: A Subanalysis

Focusing on 68/138 (49%) patients with a positive PET/CT scan, [^18^F]fluciclovine PET/CT detected a significantly higher number of recurrent lesions (83 vs. 47) compared to [^18^F]fluorocholine PET/CT (*p* = 0.002). This difference is mainly due to a greater number of lesions detected in the prostate/prostatectomy bed region (38 vs. 11, *p* < 0.0001).

Even though not statistically significant, bone was the only region with a higher number of recurrent lesions detected in the [^18^F]fluorocholine group compared to [^18^F]fluciclovine one (16 vs. 11, *p* = 0.533).

Finally, the PSA value and PSAdt significantly impacted the [^18^F]fluorocholine PET/CT lesions number, characterized by a positive linear correlation with the PSA value (*p* = 0.004) and PSAdt (*p* = 0.010). Although not statistically significant, the same trend was observed in the [^18^F]fluciclovine PET/CT group (*p* > 0.05).

### 3.5. Clinical Cases

**Figure 3:**
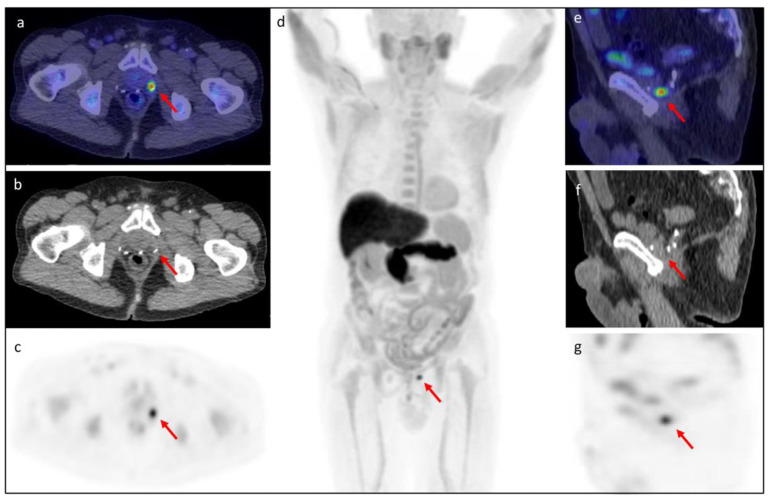
[^18^F]fluciclovine PET/CT performed in a 75-year-old patient with BCR after radical prostatectomy (Gleason Score 7 = 4 + 3). At the time of the PET/CT, PSA rose to 0.89 ng/mL with PSAdt of 7.8 months. The (**d**) MIP, (**a**–**c**) axial (**a**: fused PET/CT, **b**: CT, **c**: PET) and (**e**–**g**) sagittal (**e**: fused PET/CT, **f**: CT, **g**: PET) images showed a locoregional recurrent lesion in left prostatectomy bed (red arrow) with SUVmax 8.6.

**Figure 4:**
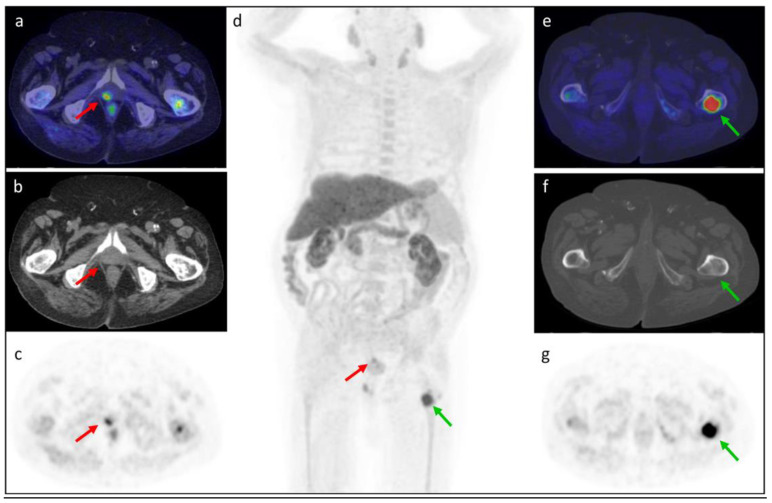
[^18^F]fluorocholine PET/CT performed in an 82-year-old patient with BCR after external radiotherapy (Gleason Score 7 = 4 + 3). PSA was 1.29 ng/mL with a PSAdt of 5.8 months. The (**d**) MIP and (**a**–**c**) axial (**a**: fused PET/CT, **b**: CT, **c**: PET) images showed a locoregional recurrent lesion in the right prostatic region (red arrow) with SUVmax 10.1. The (**d**) MIP and (**e**–**g**) axial (**e**: fused PET/CT, **f**: CT, **g**: PET) images showed a bone metastasis in the proximal epiphysis of the left femur (green arrow) with SUVmax 16.2.

**Figure 5:**
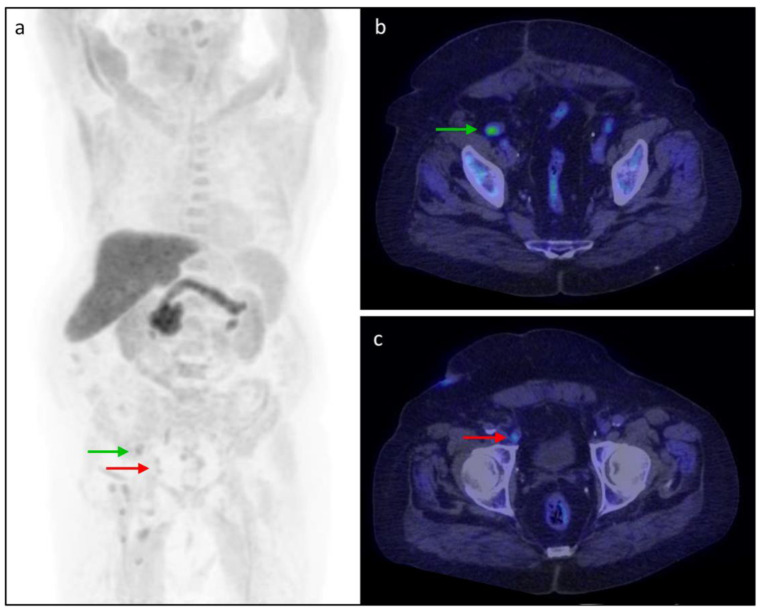
An 81-year-old patient with BCR after prostatectomy and Gleason Score 6 (3 + 3) underwent the [^18^F]fluciclovine PET/CT. PSA increased to 2.15 ng/mL with PSAdt of 13.6 months. The (**a**) MIP and (**b**,**c**) axial fused images showed recurrent lymph node lesions, namely (**b**) a right external iliac lymph node with SUVmax 5.6 (green arrow) and (**c**) a right deep inguinal lymph node with SUVmax 5.2 (red arrow).

**Figure 6:**
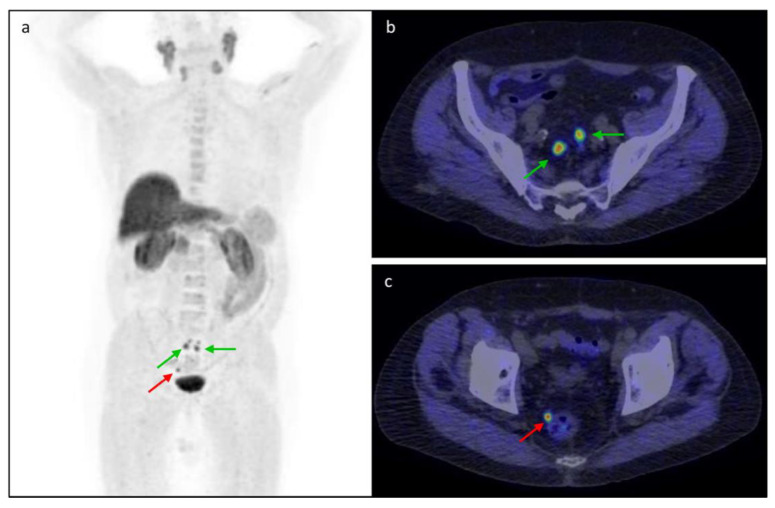
An 84-year-old patient with PSA increase (2.97 ng/mL) and low PSAdt (3.5 months) after prostatectomy (Gleason Score 8 = 4 + 4) was evaluated with the [^18^F]fluorocholine PET/CT. The (**a**) MIP and (**b**,**c**) axial fused images showed recurrent lymph node lesions, namely (**b**) two internal iliac nodes with SUVmax 8.9 (green arrow) and (**c**) one right perirectal node with SUVmax 10.3 (red arrow).

In Figure 3, Figure 4, Figure 5 and Figure 6, representative clinical cases of our sample are reported.

## 4. Discussion

The cornerstone of BCR PCa patients management is the detection of disease relapse as early as possible for correctly selecting a local or systemic therapeutic strategy. For this purpose, the limited performance of conventional imaging has been overcome by PET/CT examination with a demonstrated superior DR for PSA level less than 2 ng/mL [17].

The most recent and updated 2022 European guidelines confirm the strategic role of PET/CT in this setting of patients. For PSA recurrence after primary treatment, radiolabeled PSMA PET/CT is recommended as the first-choice imaging method, when PSA is >0.2 ng/mL. Both radiolabeled choline and fluciclovine can be considered for possible identification of disease recurrence, in case of unavailability of PSMA, for PSA ≥1 ng/mL and if the imaging result will influence subsequent treatment decisions [5].

These recommendations are consequent to the known influence of PSA level and kinetic on choline DR and sensitivity, particularly dropping to suboptimal for low PSA values. Previous studies reported a radiolabeled choline PET/CT DR of 67–100% for PSA >5 ng/mL, which decreases to 5–24% when PSA is <1 ng/mL [18]. Despite this limitation, choline PET/CT demonstrated a pooled sensitivity and specificity of 86–89% and 89–93%, respectively, and a higher specificity in bone metastases detection compared to bone scan (98–100% vs. 75–100%) [8,19,20]. These data support choline use in this setting of patients, especially for PSA values over than 1 ng/mL [5,21]. Our results are consistent with the literature data, confirming the impact of PSA value and kinetic on the [^18^F]fluorocholine PET/CT DR, which are 16% and 31% for PSA < 0.5 ng/mL and 0.5–1 ng/mL, respectively, and 21% for slower PSAdt (>12 months). The optimal PSA cut-off value of 0.94 ng/mL supports the recommendation of this radiotracer for PSA >1 ng/mL.

If the choice of [^18^F]fluorocholine as a PET radiopharmaceutical is well-established, the use of [^18^F]fluciclovine in clinical practice needs to be further investigated. Namely, our study aimed to compare the two [^18^F]-labeled radiotracers DR, in order to guide the radiopharmaceutical choice based on a specific biochemical and clinical-histological patients’ profile.

After FDA approval in May 2016, [^18^F]fluciclovine became widely commercially available and it gained a growing scientific interest for its promising role in detecting local recurrent lesions. However, the literature data concerning its diagnostic performance in BCR patients with low PSA values are still limited and conflicting. In the largest cohort study (596 patients), Bach-Gansmo et al. reported a patient-level DR of 67.7%, with a lower DR of 41.4% for PSA values ≤0.79 ng/mL [22]. A similar pooled DR (65%) was observed by Dreyfuss et al. in a 328 PCa patient cohort but preserving an optimal DR (58%) also among 26 patients with PSA < 0.2 ng/mL [23]. Analogously, Marcus et al. found an overall DR of 58% in a population including 64 PCa patients with very low PSA value (≤0.3 ng/mL) [24]. These data were also recently confirmed in the retrospective study by Filippi et al., recording a promising performance for low PSA values (DR of 66.7% for PSA <0.57 ng/mL and of 71.4% for PSA 0.57–0.99 ng/mL). In addition, the authors identified 1.1 ng/mL as the optimal cut-off value in predicting a positive PET/CT scan [25]. The minimal PSA threshold to improve the [^18^F]fluciclovine PET/CT DR in BCR PCa patients was already investigated by Armstrong et al., who suggested a PSA cut-off of 2.10 ng/mL [26]. Conversely, Wang et al. recommended performing [^18^F]fluciclovine PET/CT in patients with PSA levels ranging from 0.3 to 1 ng/mL [27]. This range includes the PSA value of 0.45 ng/mL, identified as the optimal cut-off value in predicting a positive PET scan in a previous study of our group and confirmed in the current analysis [9]. Despite the different PSA thresholds proposed, most authors agree in considering [^18^F]fluciclovine PET/CT as a reliable method also for PSA lower than the recommended EAU guideline value of 1 ng/mL.

In addition, the good diagnostic performance of the [^18^F]fluciclovine PET/CT for a low PSA value (DR 43% for PSA < 0.5 ng/mL; 61% for PSA 0.5–1 ng/mL), highlighted by the literature data and confirmed in our study, should lead us to re-evaluate the current clinical recommendations for a more precise and specific application of PET prostate-radiotracers. Hence, to guide a personalized choice of the most adequate radiopharmaceutical, it is of utmost importance to compare the diagnostic performance of the [^18^F]fluciclovine and [^18^F]fluorocholine PET/CT, the latter representing the standard of reference for PCa imaging in the last decades.

To the best of our knowledge, the literature reported only a few head-to-head comparative studies between choline labeled with [^11^C] and [^18^F]fluciclovine PET/CT diagnostic performance in small BCR patient populations [28,29,30]. The authors reported a higher [^18^F]fluciclovine DR of 20%-40% at patient-based and approximately 60% at lesion-based analysis compared to [^11^C]choline, due to a negligible urinary excretion and a high tumor-to-background ratio of the amino acid tracer [30].

However, despite the advantage of a lower rate of radioactivity in the bladder after [^11^C]choline administration, [^18^F]fluorocholine has a more widespread employment thanks to more favorable and lower positron energy (252 vs. 390 MeV) and positron range (0.66 vs. 1.27 mm), and a longer physical half-life (109.8 vs. 20.3 min), allowing its use in many PET centers without on-site cyclotron [28].

To date, there are only a few meta-analyses and no research studies aiming to directly compare [^18^F]fluciclovine PET/CT and [^18^F]fluorocholine diagnostic performance. Wang et al. reported a higher [^18^F]fluciclovine PET/CT pooled DR compared to [^18^F]fluorocholine PET/CT (74% vs. 66%). Furthermore, according to PSA categorization, the results were controversial. In fact, [^18^F]fluorocholine showed a better DR for PSA < 0.5 ng/mL (35% vs. 23%) and for PSA 1.0–1.99 ng/mL (62% vs. 57%). By contrast, the [^18^F]fluciclovine DR was higher for PSA 0.5–0.99 ng/mL (46% vs. 41%), and for PSA > 2 ng/mL (94% vs. 80%) [31]. Ma et al. calculated the DR in early disease relapse with PSA < 2 ng/mL, using choline, fluciclovine, and PSMA. Their results differed from Wang et al. study for the higher [^18^F]fluciclovine PET/CT DR compared to [^18^F]/[^11^C]choline both for low and high PSA levels, particularly 37% vs. 24% for PSA < 0.5 ng/mL, 60% vs. 36% for PSA 0.5–0.9 ng/mL, and 80% vs. 61% for PSA 1–1.99 ng/mL, respectively [32].

In our study, [^18^F]fluciclovine showed a superior overall diagnostic performance compared to [^18^F]fluorocholine PET/CT. First, the amino acid radiopharmaceutical proved to be significantly superior for the per-patient DR (64% vs. 35%) and for the number of detected recurrent lesions (83 vs. 47). Namely, this different performance appeared very relevant in the prostatic region (DR: 51% vs. 15%; the number of lesions: 38 vs. 11). Indeed, the absence of [^18^F]fluciclovine activity in the bladder at the time of image acquisition reduced the risk of masking very small locoregional recurrent sites.

Moreover, even though not statistically significant, it should be noted that the [^18^F]fluorocholine PET/CT detected a higher number of bone recurrent lesions (+13%). Considering that the [^18^F]fluciclovine bone marrow background could affect the skeletal metastases evaluation, the bone region study should be better performed using [^18^F]fluorocholine PET/CT.

Regarding the impact of different biochemical parameters on prostate-radiopharmaceuticals DR, the [^18^F]fluciclovine PET/CT was superior both for low (0.5–1 ng/mL) and for high PSA levels (>1 ng/mL). Particularly, the results for the PSA range 0.5–1 ng/mL deserve consideration, since [^18^F]fluciclovine could be very promising (DR +40%) also for PSA < 1 ng/mL. These data were confirmed in prostatic region analysis, both for low (0.5–1 ng/mL, +32%) and, especially, for high PSA levels (>1 ng/mL, +57%), further validating the greater performance in prostate/prostatectomy bed lesions detection.

Unlike [^18^F]fluorocholine, PSAdt did not significantly impact the [^18^F]fluciclovine DR. Consequently, [^18^F]fluorocholine could be inadequate for disease recurrence detection in slower PSA kinetic patients, while the [^18^F]fluciclovine PET/CT preserves a good performance also for PSAdt > 12 months (+45%).

The [^18^F]fluciclovine PET/CT superiority was also evidenced for low GS (+36%) and for the low EAU BCR risk group (+44%). In these cases, the small amount of [^18^F]fluorocholine uptake could be explained by a slow membrane metabolism, reflecting the weak proliferation of PCa cells, and by the different [^18^F]fluciclovine mechanism of uptake. Therefore, the [^18^F]fluciclovine PET/CT could prove to be very reliable imaging in low-risk patients, allowing accurate detection of the T and N parameters.

Taking into account that a precise and early detection of the recurrent site is imperative to correctly guide treatment strategies, the availability of an effective imaging modality in the case of low PSA values is crucial for optimal patient management [26].

Lastly, even if PSMA binding ligands have generally shown higher diagnostic performance when compared to other radiotracers in biochemical recurrence, the [^18^F]fluciclovine PET/CT appeared a more accurate method in detecting localized recurrence close to the bladder, as PSMA urinary activity may mask avid lesions [33]. Furthermore, [^18^F]fluciclovine could represent a promising alternative, especially for tumors characterized by PSMA-expression heterogeneity and for the non-negligible percentage of PSMA-negative patients [34].

Our study had some limitations: first, the unavailability of the histopathological confirmation of positive PET/CT, but consistent with clinical practice; second, the lack of comparative data between the [^18^F]fluciclovine and [^18^F]fluorocholine PET/CT about sensitivity, specificity, and their impact on patient management, because follow-up is still ongoing; third, the execution of the [^18^F]fluciclovine or [^18^F]fluorocholine PET/CT in our sample may have influenced the results of our study since the two radiopharmaceuticals have a different metabolism.

## 5. Conclusions

An accurate choice of the most adequate radiotracer for detecting PCa relapse should be tailored to patient profile, considering clinical, biochemical, and histological parameters.

Our comparative analysis demonstrated a better [^18^F]fluciclovine PET/CT DR compared to [^18^F]fluorocholine PET/CT for restaging PCa patients in early BCR, particularly in the detection of locoregional recurrence, also due to a negligible urinary excretion. The significantly higher [^18^F]fluciclovine DR for low PSA values supports its use in clinical practice in this setting of patients (PSA < 1 ng/mL).

This personalized approach allows for improved assessment of PCa recurrent disease, thanks to an earlier and more precise lesions detection, consequently optimizing patients management.

## Figures and Tables

**Table 1:** Patients’ characteristics (*n* = 138).

Patients’ Characteristics				
Variables	Total (*n* = 138)	[^18^F]fluorocholine (*n* = 69)	[^18^F]fluciclovine (*n* = 69)	*p*-Value
Age—y				
*Mean ± SD*	71.22 ± 6.72	71.80 ± 6.46	70.65 ± 6.96	0.705
*Median (Range)*	71.50 (50–87)	72 (52–87)	71 (50–83)	
PSA—ng/mL				
*Mean ± SD*	1.20 ± 1.17	1.25 ± 1.22	1.17 ± 1.11	0.244
PSA value—no. (%)				
*<0.5* ng/mL	49 (36%)	25 (36%)	23 (34%)	0.508
*0.5–1* ng/mL	36 (26%)	16 (23%)	21 (30%)	
*>1* ng/mL	53 (38%)	28 (41%)	25 (36%)	
PSAdt—months				
*Mean ± SD*	37.22 ± 221.03	12.92 ± 13.70	50.14 ± 273.70	0.131
*Median (Range)*	10.70 (0.10–2241.10)	8.20 (0.10–70.90)	10.75 (1.10–2241.10)	
PSAdt—no. (%)				
*≤12 months*	58 (51%)	22 (48%)	36 (53%)	0.356
*>12 months*	56 (49%)	24 (52%)	32 (47%)	
GS—no. (%)				
*<8*	96 (70%)	49 (71%)	47 (68%)	0.427
*≥8*	42 (30%)	20 (29%)	22 (32%)	
EAU BCR risk group—no. (%)				
*Low*	65 (47%)	38 (55%)	27 (39%)	0.060
*High*	73 (53%)	31 (45%)	42 (61%)	
Primary Treatment—no. (%)				
*Prostatectomy Only*	77 (58%)	42 (61%)	35 (55%)	0.105
*Radiotherapy Only*	13 (10%)	9 (13%)	4 (6%)	
*Prostatectomy + Radiotherapy*	43 (32%)	18 (26%)	25 (39%)	
Ongoing HT—no. (%)				
*Yes*	29 (21%)	17 (25%)	12 (17%)	0.202
*No*	109 (79%)	52 (75%)	57 (83%)	

Abbreviations: PSA, Prostate-Specific Antigen; PSAdt, Prostate-Specific Antigen doubling time; GS, Gleason Score; EAU, European Association of Urology; BCR, biochemical recurrence; HT, hormonal therapy.

**Table 2:** Per-patient and per-region detection rate (DR) analysis comparing the [^18^F]fluorocholine and [^18^F]fluciclovine groups stratified according to Prostate-Specific Antigen (PSA) level.

**Per-Patient Analysis**			
**PSA level**	**[^18^F]fluorocholine DR**	**[^18^F]fluciclovine DR**	***p*-value**
**<0.5 ng/mL**	5/26 (19%)	10/23 (43%)	0.063
**0.5–1 ng/mL**	4/15 (27%)	14/21 (67%)	**0.018**
**>1 ng/mL**	15/28 (54%)	20/25 (80%)	**0.040**
**Per-Region Analysis: Prostate/Prostate bed**			
**PSA level**	**[^18^F]fluorocholine DR**	**[^18^F]fluciclovine DR**	***p*-value**
**<0.5 ng/mL**	4/26 (15%)	7/23 (30%)	0.180
**0.5–1 ng/mL**	3/15 (20%)	11/21 (52%)	**0.049**
**>1 ng/mL**	3/28 (11%)	17/25 (68%)	**<0.0001**
**Per-Region Analysis: Lymph Node**			
**PSA level**	**[^18^F]fluorocholine DR**	**[^18^F]fluciclovine DR**	***p*-value**
**<0.5 ng/mL**	1/26 (4%)	5/23 (22%)	0.057
**0.5–1 ng/mL**	1/15 (7%)	5/21 (24%)	0.174
**>1 ng/mL**	7/28 (25%)	9/25 (36%)	0.284
**Per-Region Analysis: Bone**			
**PSA level**	**[^18^F]fluorocholine DR**	**[^18^F]fluciclovine DR**	***p*-value**
**<0.5 ng/mL**	1/26 (4%)	1/23 (4%)	0.724
**0.5–1 ng/mL**	0/15 (0%)	2/21 (10%)	0.219
**>1 ng/mL**	6/28 (21%)	2/25 (8%)	0.164

Bold: statistically significant

**Table 3:** Per-patient detection rate (DR) analysis comparing the [^18^F]fluorocholine and [^18^F]fluciclovine groups stratified according to Prostate-Specific Antigen doubling time (PSAdt) value.

Per-Patient Analysis			
PSA_doubling time_	[^18^F]fluorocholine DR	[^18^F]fluciclovine DR	*p*-value
**≤12 months**	12/22 (55%)	22/36 (61%)	0.412
**>12 months**	5/24 (21%)	21/32 (66%)	**0.001**

Bold: statistically significant

**Table 4:** Per-patient detection rate (DR) analysis comparing the [^18^F]fluorocholine and [^18^F]fluciclovine groups stratified according to Gleason Score, European Association of Urology (EAU) biochemical recurrence (BCR) risk group, and ongoing hormonal therapy (HT).

Per-Patient Analysis			
Gleason Score	[^18^F]fluorocholine DR	[^18^F]fluciclovine DR	*p*-value
**<8**	14/49 (29%)	30/46 (65%)	**<0.0001**
**≥8**	10/20 (50%)	14/23 (61%)	0.342
**EAU BCR Risk Group**			
**Low**	9/36 (25%)	18/26 (69%)	**0.001**
**High**	15/33 (45%)	26/43 (60%)	0.143
**Ongoing HT**			
**Yes**	9/17 (53%)	7/12 (58%)	0.537
**No**	15/52 (29%)	37/57 (65%)	**<0.0001**

Bold: statistically significant

## Data Availability

Study data are available from the corresponding author on request.
